# HTLV infection in Brazil’s second-largest indigenous reserve

**DOI:** 10.1038/s41598-022-21086-7

**Published:** 2022-10-06

**Authors:** Carolina Amianti, Larissa Melo Bandeira, Gabriela Alves Cesar, Sabrina Weis-Torres, Tayana Serpa Ortiz Tanaka, Indianara Ramires Machado, Crhistinne Cavalheiro Maymone Gonçalves, Simone Simionatto, Erica Cristina dos Santos Schnaufer, Felipe Bonfim Freitas, Antonio Carlos Rosário Vallinoto, Julio Croda, Ana Rita Coimbra Motta-Castro

**Affiliations:** 1 Universidade Federal de Mato Grosso do Sul, Campo Grande, MS Brazil; 2 Faculdade de Medicina, Universidade de São Paulo, São Paulo, SP Brazil; 3 Secretaria de Estado de Saúde de Mato Grosso do Sul, Campo Grande, Mato Grosso do Sul Brazil; 4 Universidade Federal da Grande Dourados, Dourados, MS Brazil; 5 Instituto Evandro Chagas, Ananindeua, PA Brazil; 6 Universidade Federal do Pará, Belém, PA Brazil; 7 Fiocruz Mato Grosso do Sul, Fundação Oswaldo Cruz/Ministério da Saúde/Brasil, Campo Grande, Brazil

**Keywords:** Epidemiology, Molecular biology, Microbiology, Virology

## Abstract

Human T-lymphotropic viruses 1 and 2 (HTLV-1/2) have a worldwide distribution. HTLV-1 has been associated with several diseases, including an aggressive malignant disease known as adult T-cell leukemia/lymphoma and a chronic inflammatory neurological disease called HTLV-1-associated myelopathy, while HTLV-2 has not been definitively associated with diseases. HTLV-2 is most prevalent in specific groups such as injecting drug users and the indigenous population. In Brazil, most studies about HTLV in indigenous are carried out in indigenous communities from the north of the country. Mato Grosso do Sul (MS), Central Brazil, has the second-largest indigenous population in Brazil. However, there is no available data about HTLV infection in this group. We conducted the first investigation of HTLV-1/2 infection prevalence in the indigenous population from Jaguapiru and Bororó villages in Dourados City, MS, to provide the prevalence and molecular characterization of HTLV. For that, a total of 1875 indigenous participated in the study. All the serum samples were screened by an enzyme-linked immunosorbent assay commercial kit for the presence of anti-HTLV-1/2 antibodies. Positive samples were confirmed by HTLV-1/2 Western Blot assay. The HTLV-1 5’LTR region was detected by nested PCR amplification and sequenced by Sanger. Most of the study population declared belonging to Guarani-Kaiowá ethnicity (69.18%), 872 (46.51%), and 1003 (53.49%) were from Jaguapiru and Bororó villages, respectively. The median age of participants was 31 years, and 74.24% were females. Two individuals were detected with HTLV-1 (0.1%; CI 95% 0.1–0.2). The phylogenetic analysis revealed that isolates belong to the Cosmopolitan subtype and the Transcontinental subgroup (HTLV-1aA). The low HTLV-1 prevalence found in this study is similar to that observed among blood donors, and pregnant populations from Mato Grosso do Sul. The absence of HTLV-2 infection among these Brazilian indigenous communities would suggest a distinct behavior pattern from other indigenous populations in Brazil.

## Introduction

The indigenous population in Brazil shows a high prevalence of sexually or vertically transmitted infections and a complex epidemiological profile^1–5^. Lower socioeconomic and educational levels associated with cultural aspects of some ethnicities as cross-breastfeeding, polygamy, unprotected sex, and interaction with non-indigenous society, may expose them to risk factors that lead to an increase in the detection rate of infectious diseases^1,3,6^. Mato Grosso do Sul (MS), Central Brazil, is the second state with the largest indigenous population in Brazil^7^. The Guarani-Kaiowá, KiniKinawa, Kadiwéu, Terena, Guató, Atikum and Ofaié are the ethnicities present in the state^8^. This indigenous reserve, with about 18,000 people and located in Dourados city, 3474.50 ha, is represented mainly by the Guarani-Kaiowá and Terena ethnicities living in the Bororó and Jaguapiru villages^9,10^.

The Human T-lymphotropic virus 1 (HTLV-1) is a retrovirus associated with adult T-cell leukemia/lymphoma (ATL), HTLV-1-associated myelopathy (HAM), and other inflammatory diseases^11,12^. It is estimated that 5–10 million people are infected by HTLV-1 worldwide. South Japan, sub-Saharan Africa, Caribbean islands, Melanesia, and South America are considered endemic regions for HTLV-1 infections^13^. HTLV-2 has no clinical manifestations well-defined and has been observed as prevalent in specific groups such as American indigenous populations and people who inject drugs (PWID)^14,15^. HTLV-1/2 are transmitted by sexual relations, transfusion/transplantation of contaminated blood/organs, and from mother to child, mainly by breastfeeding^16^.

HTLV-2 is predominant in the Brazilian indigenous population, which the prevalence can reach rates greater than 30%, mainly in the Kayapo Indigenous communities from the north of the country^13,17–19^. However, HTLV-1 infection can be found in this group in lower prevalence or in isolated cases^13,17,20^.

Once remaining gaps in the knowledge about HTLV infection in the country, especially among these indigenous people, this cross-sectional study aimed to investigate the prevalence, risk factors, and molecular characterization of HTLV among this second-largest indigenous reserve in Brazil located in Mato Grosso do Sul.

## Materials and methods

### Study population

**Figure 1:**
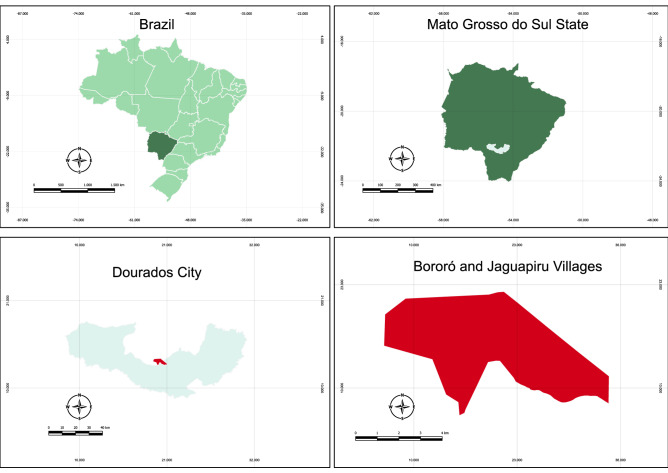
Map of the Central Brazil region showing the geographic location of the Bororó and Jaguapiru villages in the Dourados city, Mato Grosso do Sul state (MS). The map was constructed using the software QGIS 3.26.2–1.

The study population was conducted among indigenous individuals in Dourados City, MS, Central Brazil, from Bororó and Jaguapiru villages (Fig. 1).

From September 2017 to March 2020, 2190 indigenous persons were invited, and 1875 agreed to participate in the study. Participants underwent an interview with a standardized questionnaire containing sociodemographic and sexual behavior information. Blood samples were collected from all subjects to perform serological tests. The inclusion criteria were being an indigenous community member from Bororó or Jaguapiru villages and aged over 18 years, unless unable to give informed written consent. All subjects gave their written informed consent to participate in the study. This study was approved by the Comissão Nacional de ética em Pesquisa (CONEP) under protocol number 2.000.496. All research was performed following relevant guidelines and regulations.

### Serological tests

All the serum samples were screened by an enzyme-linked immunosorbent assay (ELISA) commercial kit for the presence of anti-HTLV-1/2 antibodies (GOLD ELISA HTLV-I/II—REM), following the manufacturer’s instructions. Positive samples were repeatedly tested and confirmed by HTLV-1/2 Western Blot (WB) assay (MP Diagnostics HTLV BLOT 2.4–Singapore). HTLV infection was defined as a repeatedly positive ELISA and positive WB.

### Molecular analysis

The HTLV molecular characterization of ELISA and WB positive samples was performed by nested polymerase chain reaction (nested PCR). To this end, DNA was extracted from the whole blood of the anti-HTLV positive participants using the QIAamp DNA Blood mini kit (QIAgen), according to the manufacturer’s instructions. Then, the amplification of a 646 bp fragment of the HTLV-1 5’LTR region was performed by nested PCR as previously reported^21^. Furthermore, the amplicons were purified using a QIA Quick Purification kit (Qiagen Inc., Maryland, USA), according to the manufacturer’s instructions. The fragments were sequenced using BigDye Terminator Cycle Sequencing Ready Reaction Kit and ABI 3500XL (Applied Biosystems, Foster City, CA, United States) by Sanger’s method.

The HTLV-1 isolates were subjected to an analysis in the BLAST (Basic Local Alignment Search Tool) after nucleotide sequencing to identify the subtype and subgroup. Nucleotide sequences were aligned and compared with 50 published HTLV-1 sequences available from GenBank with reference sequences used as outgroup, and sequences were searched with filter HTLV-1 and Transcontinental and Brazil using MAFFT v.7 (Multiple Alignment Program for Amino acid or nucleotide sequences). Phylogenetic analysis was performed using Maximum likelihood with 1000 bootstrap replicates with RAxML program and Bayesian method based on appropriate models determined by JModelTest (TIM2 + G model) with MR-base program on CYPRES Science Gateway. The phylogenetic tree was constructed by use of the FigTree v.1.4.4 program. The haplotype network was developed with NETWORK v.10.2 using the Median Joining algorithm.

The GenBank nucleotide sequences accession number included in the phylogenetic analysis: Z32527, L02534, L76310, Y17014, KM023757, KM023765, D13784, AF033817, DQ235699, DQ235698, Y16481, AF054627, J02029, KY510690, KY510691, KY490575, KY490581, GQ443755-57, L36905, JF271836-38, JF271840-42, AY499185, AY920503, DQ471187-97, DQ70891-92, EU392159-60, FJ853490-91, OK247616-17, KM023763-62.

### Statistical analysis

The variables of interest were analyzed using STATA 13.0 software (Stata Corporation, College Station, TX, USA). Prevalence for serological markers of HTLV infection was estimated with 95% confidence intervals. Categorical variables were presented by absolute and percentage frequency. Continuous variables were expressed as mean, standard deviation, median, and range. The chi-square test was used to evaluate differences between proportions.

### Ethics statement

The study involving human participants was reviewed and approved by the Comissão Nacional de Ética em Pesquisa (CONEP), an ethics Committee on Human Research, protocol number 2.000.496. The participants provided their written informed consent to participate in this study.

## Results

A total of 1875 individuals were enrolled in the study. The median age of participants was 31 years, including 483 males (25.76%) and 1392 females (74.24%). Regarding the village, 872 (46.51%) and 1003 (53.49%) were from Jaguapiru and Bororó, respectively. Most of them declared belonging to Guarani-Kaiowá ethnicity (69.18%). In relation to formal education, 66.28% had more than 5 years of study.

History of alcohol, drug, and sexually transmitted infections was reported by 27.68%, 3.73%, and 2.88% of the participants, respectively. None of them reported injectable drug use history. Other risk behaviors such as multiple sexual partners, irregular condom use, history of blood transfusion, tattoos, sharing sharp objects, and history of surgery are shown in Table 1.

**Table 1:** Characteristics of 1875 indigenous people of the study.

Variable	Total (%)
**Age median (years)**	31
**Gender**	
Men	483 (25.76)
Women	1392 (74.24)
**Village**	
Jaguapiru	872 (46.51)
Bororó	1003 (53.49)
**Ethnicity**	
Guarani-Kaiowá	1282 (69.18)
Guarani-Nhandeva	45 (2.43)
Terena	333 (17.97)
Other	193 (10.42)
**Study years**	
Illiterate	153 (8.43)
1–4 study years	459 (25.29)
5–12 study years	1096 (60.39)
> 12 study years	107 (5.89)
**Familiar monthly incomes**	
Less than 1 minimum wage*	952 (52.71)
1–2 minimum wages	729 (40.37)
3 or more	125 (6.92)
**People sharing the same house**	
3–5 people	1130 (60.27)
6–7 people	341 (18.18)
Other compositions	404 (21.55)
**Drug/alcohol history**	
Alcohol use	519 (27.68)
Drug use	70 (3.73)
Injectable drug use	0 (0)
**Steady sexual partner**	
No	529 (28.21)
Yes	1346 (71.79)
**Sexual history**	
Exchanged sex for money	22 (1.17)
Previously had homosexual contact	48 (2.56)
Sexual intercourse with a non-injectable drug user	143 (7.63)
Sexual intercourse with persons who inject drugs	19 (1.01)
Sexual intercourse with HIV carrier	7 (0.37)
Sexual intercourse with syphilis carrier	22 (1.17)
Sexual intercourse with hepatitis carrier	5 (0.27)
STI history	54 (2.88)
**Number of sexual partners within 5 years**	
None	189 (10.34)
1	1337 (73.18)
2 or more	301 (16.48)
**Condom use**	
Always	272 (14.51)
Sometimes/never	1603 (85.49)
**Other risk behaviors**	
History of blood transfusion	188 (10.03)
History of tattoos	457 (24.37)
History of piercing	67 (3.57)
Sharing of syringes and/or needles	30 (1.60)
History of surgery	594 (31.68)
Sharing of personal sharp objects	203 (10.83)
Previous incarceration	59 (3.15)

*The national minimum wage at the time converted to dollar was 190.00 USD.

^a^The total represents the number of individuals who answered the question**.** Percentages were calculated excluding missing data.

Anti-HTLV was detected in two individuals (0.1%; CI 95% 0.1–0.2). The two ELISA reactive samples were further tested by WB assay and confirmed anti-HTLV reactivity with complete profiles in the WB test for HTLV-1. The test results were subsequently referred to the health authorities responsible for this study population.

**Figure 2:**
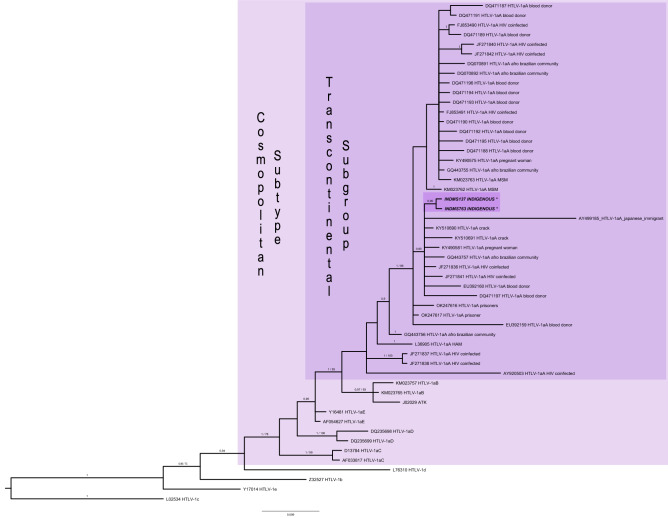
Phylogenetic tree of HTLV-1 subtypes from different groups. Phylogenetic tree constructed based on Maximum likelihood and Bayesian method with sequences of HTLV-1 5’LTR region from indigenous of this study (highlighted) and using 50 sequences from GenBank. Support for the branching was determined by 1000 bootstrap replicates and only values of 70% or superior was shown for Maximum likelihood and 0.9 or superior of posterior probability for Bayesian analyses. ID-137 (OM863789), ID-763 (OM863790) clustered with different population groups.

The HTLV-1 5’LTR region was detected by nested PCR amplification in the two anti-HTLV-1 positive samples, which were successfully sequenced. The phylogenetic analysis revealed that both isolates were classified as belonging to the Cosmopolitan (1a) subtype and the Transcontinental (A) subgroup (Fig. 2). The GenBank accession numbers for the sequences were: OM863789 (ID-137) and OM863790 (ID-763). Due to low variation between the sequences, a haplotype network with HTLV-1aA sequences (Supplementary Fig. S1) was performed to show the differences between sequences.

Table 2 shows the sociodemographic characteristics and risk behaviors of the two anti-HTLV positive individuals. One of the positive individuals, a 42-year-old female (ID-763), was married twice. No information was available on the HTLV-1 status of her first husband, with whom she had 6 children. Also, she had 1 daughter with her current husband. Of these 6 older children, 3 were found at the time of our visit and referred to the health authorities responsible. The current husband did not agree to participate in the study. Besides, their daughter was under 2 years old and was being breastfed by her mother. She reported having breastfed four of her children for more than 1 year and the other two children for less than 6 months. She also reported having normal labor and that her tested offspring were negative for the presence of anti-HTLV-1/2 by the ELISA method.

**Table 2:** Sociodemographic and risk behavior characteristics of the two HTLV-1 infected individuals.

Characteristics	ID-137	ID-763
HTLV	HTLV-1aA	HTLV-1aA
Age (years)	81	42
Study years	6	5
Marital status	Not informed	Married
Gender	Male	Female
Ethnicity	Guarani-Kaiowá	Guarani-Kaiowá
Village	Bororó	Bororó
Naturality	Dourados-MS	Dourados-MS
IDU history	No	No
Blood transfusion before 1993	No	No
Condom use	Sometimes/never	Sometimes/never
Sexual preference	Heterosexual	Heterosexual
Previous incarceration	No	No
Number of sexual partners within 5 years	0	1
Previous HIV, viral hepatitis, and Syphilis test	No	Yes (negative results)

ID-identification number of the participant sample, IDU-Injection drug use, MS-Mato Grosso do Sul state.

The other positive individual was an 81-year-old man (ID-137). Both individual ID-137 and ID-763 did not report any risk behaviors such as injection drug use, unprotected sexual intercourse, and multiple sexual partners. Both positive individuals were born in Dourados city, lived in Bororó village, and declared themselves Guarani-Kaiwá ethnicity.

## Discussion

This is the first investigation of HTLV-1/2 infection prevalence carried out among the indigenous population from Central Brazil. The prevalence of HTLV infection found in this study (0.1%; CI 95% 0.1–0.2) is similar to the prevalence observed in the blood donors, and pregnant population from Mato Grosso do Sul (0.2% and 0.1%, respectively)^22,23^. However, the results of this present study contrast with most of the studies conducted among indigenous populations in Brazil.

Several studies carried out among the indigenous population from the Brazilian Amazon region were developed, and HTLV-2 infection was shown to be prevalent, which is a change in the epidemiological pattern of this HTLV type in this specific group^1,24,25^. The high HTLV-2 prevalence in the indigenous population in Brazil usually differs between communities (from 1.4% in the Wayampi community on Amapá to 57.9% in the Kayapo community on Pará)^1,13,17,24,26^. Unlike these studies conducted in Brazil, the present study, no individuals infected with HTLV-2 were found.

The introduction of HTLV-1 in the Mato Grosso do Sul State probably occurred during the human migratory process from the slave trade from Africa and Japanese migration in the previous centuries^27–29^. A prevalence study conducted among several Brazilian indigenous populations has already identified HTLV-1 infection, and all of them were from the North region of the country. Ishak et al.^17^ observed HTLV-1 infection prevalence of 2.94%, 0.67%, and 0.48% among Yanomami, Galibi, and Aukre communities, respectively. Besides, a prevalence of 0.62% was found in the Wayampi community in 2002, and one case was reported in the same community in 2007^20,30^. The absence of more recent HTLV-1 studies in a Brazilian indigenous group may be due to the cultural characteristics of the studied communities since most studies are carried out in closed communities^27^. The geographic isolation of these villages limits contact with urban centers or other indigenous groups^24^. However, the Jaguapiru and Bororó villages are located 5 km from Dourados city, the second-largest city of Mato Grosso do Sul state, therefore geographically accessible to the urban center^7,31,32^. HTLV-1 infection was recently reported among Warao indigenous refugees living in urban area of Belém city^33^. Also, HTLV-1 is already reported in Indigenous people from other South American countries, such as Chile and Venezuela^13,33–35^. There was no difference of positivity for anti-HTLV (0.10% vs. 0.20%; *p* > 0.30) between female and male participants, respectively. These results are in accordance with studies conducted among the indigenous population in which no difference between genders was found^17,36^.

HTLV-1 testing has been included in the routine antenatal screening of the state of Mato Grosso do Sul by the Program for Protection of Pregnant Woman since 2002^23^. The positive female individual (ID-763) reported that she was evaluated during her youngest daughter's pregnancy by the antenatal screening. She was diagnosed with positive anti-HTLV-1/2 and was recommended the interruption of breastfeeding since that time. The Brazilian Ministry of Health recommends the interruption of breastfeeding and the provision of milk formula substitutes feeding for those babies born from HTLV-1/2 seropositive mothers to avoid mother-to-child transmission^16^. Although these measures, adequate counseling with information about HTLV-1 transmission or clinical evaluation to identify signs or symptoms of HTLV-1 associated diseases was not offered to the ID-763 participant. This demonstrates the need to spread knowledge about HTLV infection and the public health policies that are already implemented in Brazil to properly provide counseling for positive patients.

The HTLV-1aA sequences present in this study diverge from the previous studies conducted in Brazil among indigenous populations that found mainly HTLV-2. The sequences obtained from the present study grouped next with other isolates from various non-indigenous populations and geographical regions in Brazil, such as people who use drugs, Afro-descendants, pregnant women, blood donors, and people who live with HIV (PLHIV), suggesting a possible HTLV-1 transmission from these non-indigenous individuals. Unfortunately, sequences from other worldwide indigenous populations belonging to the Transcontinental subgroup (HTLV-1aA) were not found registered on Genbank.

Some limitations must be acknowledged. First, underreporting of risk behaviors and reporting bias are some of the limitations of this cross-sectional study. Because some of them may have been omitted during the interviews due to the distrust of discrimination and stigma. In addition, individuals from the micro-areas of indigenous health care were enrolled by convenience sampling, with greater acceptance of the study among residents of the Bororó village, resulting in unequal sample distribution between them. Because of the low number of HTLV infected individuals in this study, regression analysis could not be performed. Despite these limitations, this study is representative of the indigenous population of Central Brazil since we used a large sample size. Moreover, our study has important information which would contribute to closing the gaps in HTLV epidemiology and controlling public policies.

The results of the study show the presence and circulation of HTLV-1 in indigenous populations from Jaguapiru and Bororó villages in Dourados City, MS. A better understanding of the patterns of occurrence of HTLV-1 in the indigenous population of Mato Grosso do Sul is critical to guiding the development of public health initiatives to reduce transmission. Although a higher risk of infection has been supposed for this specific study population, the prevalence rate of HTLV-1 infection found in this study was similar to that observed among Brazilian blood donors (0.48%)^37^. Also, the absence of HTLV-2 infection among these Brazilian indigenous communities would suggest a behavior pattern distinct from other indigenous populations in Brazil.

## Supplementary Information

Supplementary Figure S1.

## Data Availability

All the relevant and original data presented in the study are included in the article. The datasets generated and/or analyzed during the current study are not included in a publicly available repository due to restrictions applied to the availability of these data, such as possible identification of the participants, being a restricted group, but are available from the corresponding author on reasonable request. DNA sequences were registered into the GenBank repository under accession numbers OM863789 (ID-137) and OM863790 (ID-763).
