# Supplementary material for: HTLV infection in Brazil’s second-largest indigenous reserve
Source: Sci Rep. 2022 Oct 6;12:16701. doi: 10.1038/s41598-022-21086-7 (PMC9537150; doi:10.1038/s41598-022-21086-7)
Supplement: Supplementary file 1 — Supplementary Figure S1. [file 41598_2022_21086_MOESM1_ESM.pdf]

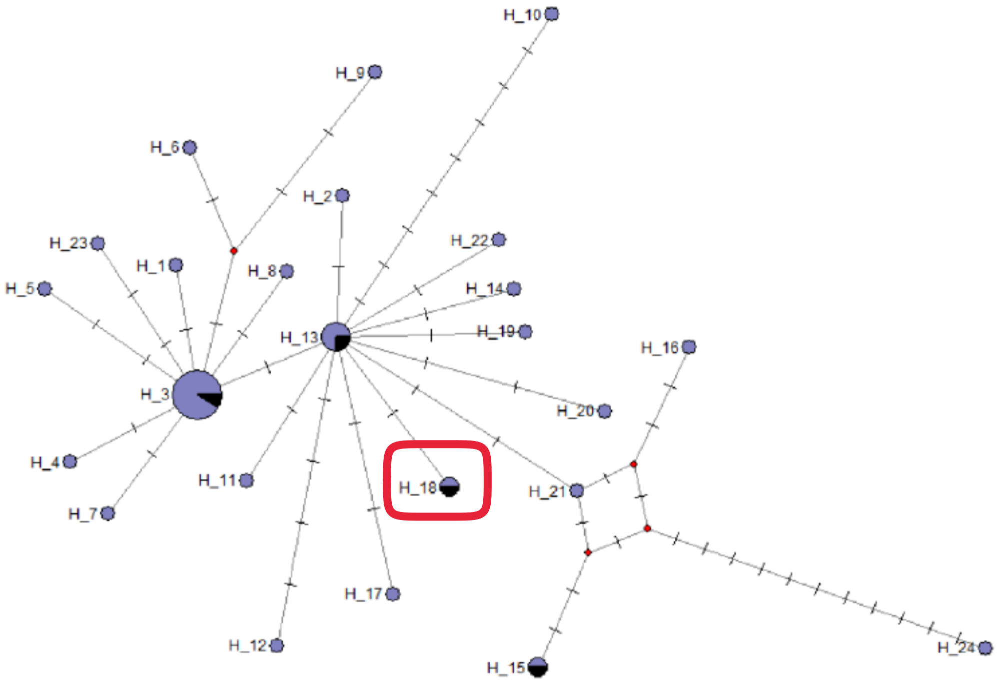

Supplementary Figure S1. Haplotype **network of HTLV-1aA from different groups.**

Haplotype network based on the total of Transcontinental (A) sequences from phylogenetic tree (39 sequences). GenBank nucleotide sequences accession number: H_1: KY490575; H_2: KY49058; H_3: FJ853491, FJ853490, DQ471190, DQ471191, DQ471196, DQ471193, JF271842, JF271840, GQ443755, KM023763, DQ070892; H_4: DQ471189; H_5: DQ471192; H_6: DQ471187; H_7: DQ471188; H_8: DQ471194; H_9: DQ471195; H_10: EU392159; H_11: EU392160; H_12: DQ471197; H_13: JF271836, KM023762, KY510690, OK247617; H_14: JF271841; H_15: JF271837, JF271838; H_16: L36905; H_17: AY499185; H_18: OM863789 (ID-137), OM863790 (ID-763); H_19: GQ443757; H_20: KY510691; H_21: GQ443756; H_22: OK247616; H_23: DQ070891; H_24: AY920503.
